# Jumping translocations of chromosome 1q occurring by a multi-stage process in an acute myeloid leukemia progressed from myelodysplastic syndrome with a *TET2* mutation

**DOI:** 10.1186/s13039-019-0460-2

**Published:** 2019-11-19

**Authors:** Ina Lee, Mary A. Gudipati, Elizabeth Waters, Vu H. Duong, Maria R. Baer, Ying Zou

**Affiliations:** 1 Department of Pathology, University of Maryland School of Medicine, Baltimore, MD USA; 2 Department of Medicine, University of Maryland School of Medicine, Baltimore, MD USA; 3 University of Maryland Marlene and Stewart Greenebaum Comprehensive Cancer Center, Baltimore, MD USA; 4 Department of Pathology, Johns Hopkins University, 1812 Ashland Ave., Suite 200, Room 221, Baltimore, MD 21205 USA

**Keywords:** Jumping translocations, Acute myeloid leukemia, Myelodysplastic syndrome, *TET2*

## Abstract

**Background:**

Jumping translocations (JTs) are rare chromosome rearrangements characterized by re-localization of one donor chromosome to multiple recipient chromosomes. Here, we describe an acute myeloid leukemia (AML) that progressed from myelodysplastic syndrome (MDS) in association with acquisition of 1q JTs. The sequence of molecular and cytogenetic changes in our patient may provide a mechanistic model for the generation of JTs in leukemia.

**Case presentation:**

A 68-year-old man presented with pancytopenia. Bone marrow aspirate and biopsy showed a hypercellular marrow with multilineage dysplasia, consistent with MDS, with no increase in blasts. Karyotype and MDS fluorescence in situ hybridization (FISH) panel were normal. Repeat bone marrow aspirate and biopsy after 8 cycles of azacitidine, with persistent pancytopenia, showed no changes in morphology, and karyotype was again normal. Myeloid mutation panel showed mutations in *RUNX1*, *SRSF2*, *ASXL1*, and *TET2*. Three years after diagnosis, he developed AML with myelodysplasia-related changes. Karyotype was abnormal, with unbalanced 1q JTs to the short arms of acrocentric chromosomes 14 and 21, leading to gain of 1q.

****Conclusions**:**

Our patient had MDS with pathogenic mutations of the *RUNX1*, *SRSF2*, *ASXL1*, and *TET2* genes and developed 1q JTs at the time of progression from MDS to AML. Our data suggest that the formation of 1q JTs involves multiple stages and may provide a mechanistic model for the generation of JTs in leukemia.

## Background

Jumping translocations (JTs) are chromosomal rearrangements comprising one donor chromosome and multiple recipient chromosomes [1]. Although JTs have been reported in neoplasms and constitutional chromosome abnormalities, they are rare chromosome rearrangements in neoplastic diseases. JTs are characterized by translocations of one donor chromosome to various recipient chromosomes, resulting in several gains of this chromosomal segment and possible loss of segments of the recipient chromosomes [1, 2]. Fusion of the break-off donor chromosome segment to telomeric or interstitial regions of recipient chromosomes can form different chromosomal patterns of jumping translocations. Jumping translocations involving 1q12–21 as the donor chromosome segment, referred to as jumping translocations of 1q (1q JTs), are nonrandomly involved in multiple myeloma and malignant lymphoproliferative disorders [3, 4]. 1q JTs have been described infrequently in patients with myeloid malignancies and have been associated with a high risk of transformation to acute myeloid leukemia (AML), resistance to chemotherapy and poor survival rates [5, 6].

While several mechanisms have been proposed to explain JT formation, including viral infection, chromosome instability, pericentromeric heterochromatin de-condensation, shortened telomeres, and illegitimate recombination between telomere repeat sequences and interstitial telomeric sequences [3, 7–13], the mechanism of 1q JT formation in patients with myeloid malignancies is still not fully understood. Here, we describe a patient with AML that progressed from a myelodysplastic syndrome (MDS) with pathogenic mutations of the *RUNX1*, *SRSF2*, *ASXL1*, and *TET2* genes in association with development of 1q JTs, which supports that the formation of 1q JTs may involve multiple stages and that 1q JTs may represent a very high-risk cytogenetic abnormality with transformation to AML.

## Case presentation

**Fig. 1:**
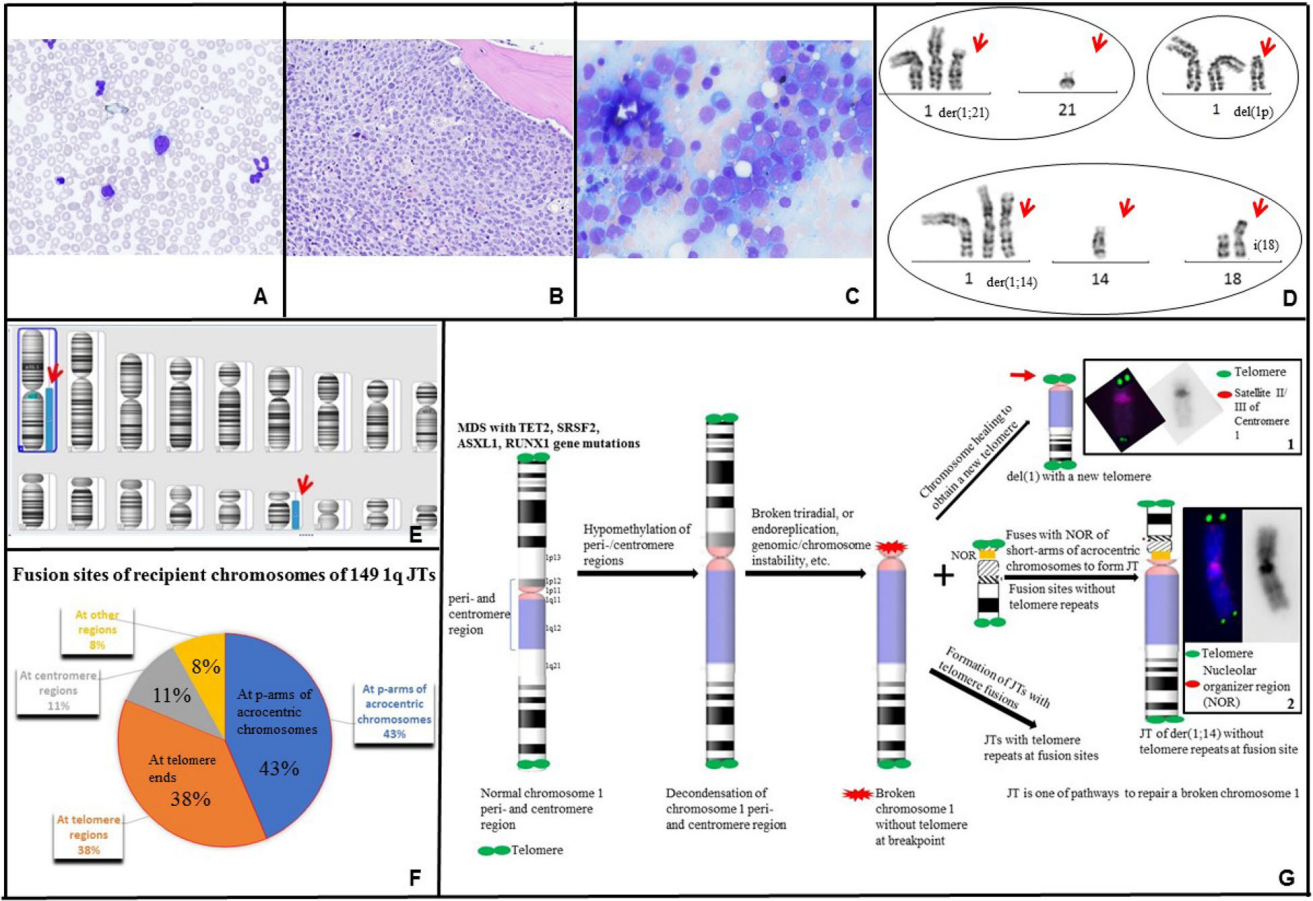
**a** Peripheral blood shows marked leukocytosis with numerous blasts and promyelocytes, dyspoietic granulocytes with nuclear hypolobation and hypogranularity, and dyspoietic erythroid precursors. **b** Bone marrow core biopsy is hypercellular for age (80%). Maturing granulopoiesis and erythropoiesis are replaced by sheets of immature cells. Megakaryocytes are decreased and have atypical morphology. **c** Bone marrow aspirate consists of blasts which are intermediate in size with fine chromatin, prominent nucleoli and scant basophilic cytoplasm. A few dyspoietic maturing granulocytes and atypical megakaryocytes are present. **d** Partial karyograms of a 46,XY,+ 1,der(1;21)(p10 or q10;q10) karyotype, a 46,XY,+ 1,del(1)(p12) karyotype, and 46,XY,+ 1,der(1;14)(p10 or q10;q10),i(18)(q10) karyotype. **e** Whole-genome SNP microarray shows mosaic gain of chromosome 1 from 1p11 to 1qter regions and mosaic gain of chromosome 18q. **f** Fusion sites of recipient chromosomes of 149 jumping translocations of 1q in 48 myeloid neoplasm patients (including our patient). **g** A possible multi-stage process for the development and formation of 1q JTs in our patient.

A 68-year-old man presented with pancytopenia. Bone marrow aspirate and biopsy showed a hypercellular marrow (90%) with multilineage dysplasia, consistent with MDS, with no increase in blasts. Karyotype and MDS fluorescence in situ hybridization (FISH) panel were normal. Repeat bone marrow aspirate and biopsy after 8 cycles of azacitidine, with persistent pancytopenia, showed no changes in morphology, and karyotype was again normal. Myeloid mutation panel showed mutations in *RUNX1* (Glu223Glyfs*16), *SRSF2* (Pro95His), *ASXL1* (Gln976*), and *TET2* (Ser890*) (TruSight myeloid sequencing panel, Illumina, Inc.). He received several other unsuccessful therapies, with serial bone marrow testing showing no change in morphology, a normal karyotype, and no change in myeloid mutations. Three years after diagnosis, his white blood cell count increased rapidly to 36.9 K/mcL with 20% blasts (Fig. 1a). Bone marrow biopsy (Fig. 1b) and aspirate (Fig. 1c) were hypercellular (80%) with increased reticulin fibrosis (Grade 2–3/3) and with 53% myeloblasts by aspirate differential, diagnostic of AML with myelodysplasia-related changes. Karyotype was abnormal, with unbalanced 1q JTs: 46,XY,+ 1,der(1;21)(p10 or q10;q10) [7]/46,XY,+ 1, der(1;14)(p10 or q10;q10),i(18)(q10) [5]/46,XY,+ 1,del(1)(p12, 1]/46,XY [8] (Fig. 1d). FISH analyses of prior bone marrow biopsies, including one obtained less than a month prior to transformation to AML, did not show 1q JTs. A week later, the patient presented to the emergency department after a fall, became obtunded, and was diagnosed with necrotizing subdural abscess and bacteremia. He was transitioned to comfort care and passed away the next day.

### Characterization of the 1q JTs in our patient

Whole-genome single nucleotide polymorphism (SNP) microarray showed mosaic gain of chromosomes 1p11-1q44 and 18q11.1-18q23, arr [hg19] 1p11q44(120,365,518_ 249,224,684)× 2–3,18q11.1q23(18, 811,960_78,014,123)× 2–3 (Fig. 1e). The 1q JTs were demonstrated to have a chromosome 1 centromere using a centromere 1 Satellite II/III FISH probe (Abbott/Vysis, Inc.), and to contain ribosomal ribonucleic acid (rRNA) genes located in nucleolar organizer regions (NORs) of short arms of the acrocentric chromosomes using an acro-p-arm probe (Abbott/Vysis, Inc.) (Fig. 1g, insertions 1–2). Telomere FISH did not show telomere repeats in fusion sites of the 1q JTs using telomere-specific (TTAGGG)_3_ probes (Applied Biosystems, Foster City, CA) (Fig. 1g, insertion 2).

### Literature review of 1q JTs in myeloid neoplasms

A literature search revealed 48 cases of myeloid neoplasms with 1q JTs (including our patient, Table 1) [5, 6, 11, 14–24]. Of 40 patients who did not have AML at the time of diagnosis, 21 (52.5%) transformed to AML and had a poor outcome. In terms of recipient chromosomes, 1q JTs in myeloid malignances have been fused to the telomere regions of recipient chromosomes in 81% of 149 1q JTs, and more than half of these fused to the short arms of the five acrocentric chromosomes in the human genome (Table 1). In terms of recipient chromosomes, among 149 1q JTs in 48 patients with myeloid neoplasms, 43% of the fusions occurred in short arms of acrocentric chromosomes, 38% occurred in telomeric regions of chromosome arms, 11% occurred in the pericentromeric/centromere regions, and 8% occurred in interstitial regions of recipient chromosomes (Fig. 1f). The most frequently seen fusions are in short arms of all five acrocentric chromosomes including 15p (12%), 14p (8.8%), 22p (8.8%), 21p (7.5%), and 13p (6.1%) (Table 1).

**Table 1:**
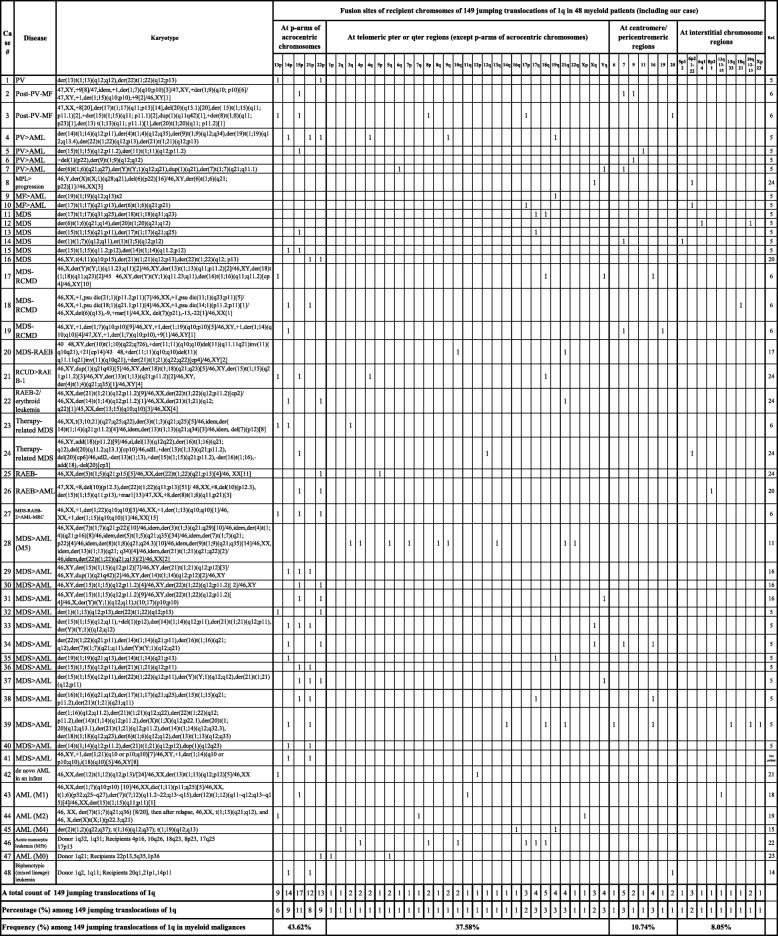
149 jumping translocations of 1q in 48 myeloid neoplasm patients (including our case**)**

## Discussion and conclusions

Our patient had MDS with pathogenic mutations of the *TET2*, *RUNX1*, *SRSF2*, and *ASXL1* genes and developed 1q JTs at the time of progression from MDS to AML. Our data suggest that the formation of 1q JTs may involve multiple stages, including pathogenic mutations of the *TET2* gene and/or other myeloid genes, hypomethylation/decondensation of the donor pericentromeric regions of chromosome 1, shortened/dysfunctional telomeres in recipient chromosomes, as well as unique structure of short arms of acrocentric chromosomes.

TET proteins, such as TET2, play key roles in the regulation of DNA-methylation status [25]. The *TET2* gene (OMIM*612839) encodes a methylcytosine dioxygenase that catalyzes the conversion of 5-methylcytosine to 5-hydroxymethylcytosine [25]. It can both serve as a stable epigenetic mark and participate in active demethylation [25]. Patients with myeloid malignancies and *TET2* mutations have a higher response rate with hypomethylating agents (such as azacitidine or decitabine) than patients who are with wild-type for *TET2* [26]. The pericentromeric heterochromatin region of chromosome 1 can become hypomethylated by in vitro modification using 5-Azacitidine [8]. The *RUNX1* gene (OMIM*151385) encodes a Runt-related transcription factor and binds to deoxyribonucleic acid (DNA) via a Runt domain. It has a primary role in the development of all hematopoietic cell types and can produce oncogenic transformation to AML. Recent data also suggested that RUNX1 contributes site specificity of DNA demethylation by recruitment of TET2 and other demethylation-related enzymes to its binding sites in hematopoietic cells [27]. The *SRSF2* gene (OMIM*600813) is a splicing factor, which is required for spliceosome assembly. It regulates constitutive and alternative splicing and maintains genome stability through the prevention of R-loop structure formation during transcription [28, 29]. The *ASXL1* gene (OMIM*612990) encodes for a chromatin-binding protein and disrupts chromatin in localized areas which leads to enhanced transcription of some genes, while repressing the transcription of others [30]. It facilitates a chromatin polycomb protein to maintain both activation and silencing of homeotic genes [31]. Through interaction with the PRC2 complex, loss of *ASXL1* results in a genome-wide reduction in H3K27 trimethylation [31]. Pathogenic mutations of the *TET2* gene along with other genes and/or treatment with azacitidine in our patient may have played a role in hypomethylation/de-condensation of pericentromeric heterochromatin of chromosome 1.

Most reported cases with 1q JTs were characterized by banding and FISH methods with fusion breakpoints on chromosome 1 mainly in its long arm (1q10-q12, 1q21), and rarely in its short arm (1p10-p11). Our patient had a pericentromeric 1p11 band in the short arm of chromosome 1 as a breakpoint of the donor chromosome of JTs. In terms of recipient chromosomes, the majority of the fusions occurred in short arms of acrocentric chromosomes (Table 1). The short arms of the five acrocentric chromosomes have a unique structure, with NORs sandwiched between centromeric and telomeric heterochromatin. Proximal (centromeric) side sequences of the NORs are almost entirely segmentally duplicated, like the regions bordering centromeres. As human NORs show enhanced instability in cancers, pericentromeric heterochromatin of chromosome 1 may fuse with similar sequences of the proximal sides of NORs. By FISH analyses, the JTs had a chromosome 1 centromere, NORs at short arms of the recipient acrocentric chromosomes, and no telomere repeats in fusion sites. Therefore, fusion sites of 1q JTs in our case had NORs, but no telomere repeats (Fig. 1g, insertion 2), which may shed light on why 43% reported 1q JTs in myeloid malignances are in the short arms of the five acrocentric chromosomes (Fig. 1f).

Telomere length has been reported to be decreased in AML cells with JTs [7] and telomere shortening, or dysfunctional telomeres may contribute to the formation of 1q JTs, which may explain why 38% of reported 1q JTs occurred in telomeric regions of chromosome arms (Fig. 1f). One cell in our patient had a deleted chromosome 1 with loss of the 1p12 - 1p36.3 segment, but had telomere repeats on both telomere ends (Fig. 1g, insertion 1), suggesting the presence of a chromosome healing event leading to addition of a new telomere onto a chromosome break.

Our data suggest that the formation of 1q JTs involves multiple stages (Fig. 1g). The leukemic process in our patient was likely initiated by pathogenic mutations in MDS/AML disease-related genes, leading to MDS. Then mutations of myeloid genes and treatment with a hypomethylating agent (such as azacitidine in our patient) may lead to hypomethylation/de-condensation of pericentromeric/centromere heterochromatin of chromosome 1, resulting in a broken chromosome 1 with a pericentromeric/centromere break. Additionally, telomere shortening/dysfunction increased susceptibility to genomic/chromosome instability. Subsequently, if the broken chromosome 1 without telomeres was not restored by a chromosome healing event by seeding a new telomere onto a chromosome break, it could be repaired by fusing with either NOR regions of acrocentric chromosomes or shortened telomere ends of recipient chromosomes (possibly through illegitimate recombination) to form 1q JTs in order to achieve their stabilization. The 1q JTs in our patient occurred in the short arms of acrocentric chromosomes 14 and 21, leading to gain of 1q. Finally, 1q JTs cells with extra copies of 1q with or without additional chromosome abnormalities may have a proliferative advantage, leading to disease progression from MDS to AML, clonal evolution and more aggressive disease. Our data may provide a mechanistic model for the generation of JTs in leukemia. Further investigation of sequences around the fusion sites would provide the molecular key to how these events are orchestrated in development and formation of JTs.

## Data Availability

All data generated or analyzed in this study are included in this published article [and its additional files].

## Abbreviations

AML: Acute myeloid leukemia
DNA: Deoxyribonucleic acid
FISH: Fluorescence in situ hybridization
JTs: Jumping translocations
MDS: Myelodysplastic syndrome
NORs: Nucleolar organizer regions
rRNA: Ribosomal ribonucleic acid
SNP: Single nucleotide polymorphism
